# Intermittent versus Continuous Catheterization and Differences in the Evolution of Labor: Systematic Review and Meta-analysis

**DOI:** 10.1055/s-0041-1740209

**Published:** 2021-12-21

**Authors:** Inês Reis, Sara Cunha, Matilde Martins, Luísa Sousa, Adérito Seixas, Cátia Rasteiro

**Affiliations:** 1 Serviço de Ginecologia e Obstetrícia do Centro Hospitalar de Entre o Douro e Vouga, Santa Maria da Feira, Portugal; 2 Escola Superior de Saúde, Fundação Fernando Pessoa, Porto, Portugal; 3 LABIOMEP, INEGI-LAETA, Faculty of Sports, University of Porto, Porto, Portugal; 4 Faculdade de Ciências da Saúde, Universidade da Beira Interior, Covilhã, Portugal

**Keywords:** labor, urinary catheter, bladder drainage, transurethral catheter, trabalho, cateter urinário, drenagem da bexiga, cateter transuretral

## Abstract

**Objective** To evaluate the differences between bladder emptying options (permanent catheterization and intermittent bladder emptying/spontaneous urination) regarding the effects on labor length, need of operative vaginal deliveries, and cesarean section rate.

**Data Sources** The search was conducted in MEDLINE, Scopus, Web of Science, and The Cochrane Central Register of Controlled Trials databases.

**Selection of Studies** The survey returned 964 studies. A total of 719 studies were evaluated by title and abstract, of which 4 were selected for inclusion.

**Data Collection** All references were inserted in the Rayyan QCRI tool (Rayyan Systems Inc., Cambridge, MA, USA). The full text of the selected articles was obtained so we could later decide whether or not to include them in this systematic review.

**Data Synthesis** No differences were found in the number of instrumented deliveries or in cesarean section rate between groups.

**Conclusions** After evaluating the studies performed on the topic, we concluded that there is no clear advantage to either method, although continuous catheterization was associated with a greater occurrence of eutocic births. In the remaining outcomes, there were no differences between catheterization types.

## Resumo

**Objetivo** Avaliar as diferenças entre as opções de esvaziamento vesical (cateterismo permanente e esvaziamento vesical intermitente/micção espontânea) em relação aos efeitos na duração do trabalho de parto, necessidade de partos vaginais operatórios e taxa de cesárea.

**Fontes de Dados** A pesquisa foi realizada nas bases de dados MEDLINE, Scopus, Web of Science, e The Cochrane Central Register of Controlled Trials.

**Seleção de Estudos** A pesquisa retornou 964 estudos. Um total de 719 estudos foram avaliados por título e resumo, dos quais 4 foram selecionados para inclusão.

**Coleta de Dados** Todas as referências foram inseridas na ferramenta Rayyan QCRI (Rayyan Systems Inc., Cambridge, MA, EUA). O texto completo dos artigos selecionados foi obtido para posterior decisão de incluí-los nesta revisão sistemática.

**Síntese dos Dados** Não foram encontradas diferenças no número de partos instrumentados ou na taxa de cesariana entre os grupos.

**Conclusões** Após avaliação dos estudos realizados sobre o tema, concluímos que não há vantagem clara de qualquer um dos métodos, embora o cateterismo contínuo tenha sido associado à maior ocorrência de partos eutócicos. Nos demais desfechos, não houve diferenças entre os tipos de cateterismo.

## Introduction

In parturients under neuraxial analgesia, the reduced perception of a full bladder and the inability to void can lead to urinary retention. These aspects, together with fluid therapy and the duration of labor, make bladder emptying a necessity. There is no consensus in the literature, or in the practice of delivery rooms, whether intermittent bladder emptying or continuous catheterization during neuraxial analgesia and until delivery is more appropriate. Therefore, the aim of the present study is to summarize and critically evaluate the evidence concerning the different bladder emptying options (permanent catheterization and intermittent bladder emptying/spontaneous urination) in terms of their effects on labor length, need of operative vaginal deliveries, and cesarean section rate. 1 2 3 4 5 6 7 8

Childbirth, as a fundamental moment in a woman's life, has been a popular subject of research over the past decades, with the introduction of methods and options that allow women to experience a less traumatic and painful experience, promoting the well-being of women and fetuses. Neuraxial analgesia is considered an integral part of normal labor, being the most effective and safe analgesia option (Petitprez et al., 2020). 5

Classically, neuraxial analgesia has been associated with an increase of cesarean section rates and operative vaginal deliveries, as well as with a longer duration of labor (referred in several protocols as an additional 1 hour in the 2 ^nd^ stage of labor period). However, recent reviews, validated in guidelines, demonstrate that this is not the case (American College of Obstetricians and Gynecologists [ACOG], 2019). 1

## Methods

This review was reported based on the Preferred Reporting Items for Systematic Reviews and Meta-Analysis (PRISMA) recommendations.

Randomized controlled studies in humans that included women in labor (induced or spontaneous) with neuraxial analgesia in which continuous versus intermittent catheterization were compared were considered for inclusion if focusing on the effects in labor length, type of delivery, and cesarean section rate. No publication date restrictions were defined. Articles published in Portuguese, English, French, Spanish, and Italian were considered.

Continuous catheterization was defined as permanent catheterization from the time of neuraxial analgesia until the 2nd stage of labor. Intermittent catheterization was considered as the introduction of a urinary catheter to empty the bladder, with immediate removal after emptying.

Neuraxial analgesia included epidural, combined (spinal-epidural), and spinal techniques.

The duration of labor was defined as the time from neuraxial analgesia to the end of the second stage of delivery and was counted in hours.

The type of delivery was classified as eutocic, operative vaginal delivery (vacuum or forceps extraction), and cesarean section.

The electronic search was conducted in MEDLINE, Scopus, Web of Science, and The Cochrane Central Register of Controlled Trials databases using the following search string ( *labor* OR *delivery* OR *parturition* OR *childbirth* ) AND ( *urethral catheter* * OR *urinary catheter* * OR *bladder drainage* OR *transurethral catheter* *).

In addition, a secondary search was conducted on the reference list of included articles to identify other possible relevant studies.

The keywords used were based on the Patient, Intervention, Comparison, and Outcome (PICO) strategy, focusing on women in labor with neuroaxis analgesia (participants) who needed bladder emptying (intervention), with a comparison between permanent and intermittent catheterization (comparison) to assess duration labor, type of delivery, and rate of cesarean sections (outcomes).

All identified references through database and reference screening (identification) were exported to the Rayyan QCRI tool (Rayyan Systems Inc., Cambridge, MA, USA), and duplicate results were removed. Initially, articles were selected by title and abstract (screening). Subsequently, the full text of the selected articles was analyzed for eligibility (eligibility), and all relevant studies were included in the systematic review. All steps were performed individually by two independent reviewers, and disagreements were resolved by consensus. The same authors were responsible for extracting data from the articles included in the review. Data related to study identification, study design, demographic data, follow-up time, intervention, and data related to childbirth and postpartum were extracted.

Two reviewers used the Cochrane tool to analyze the risk of bias in randomized trials (RoB2).

Data extracted by both researchers were inputted in the RevMan software, version 5.3 (The Cochrane Collaboration, London, UK). Heterogeneity was assessed from a methodological (methodology of the studies), clinical (clinical characteristics of the sample), and statistical (calculation of the I ^2^ value) perspective. Given the methodological and clinical heterogeneity of the studies, the random-effects meta-analysis model (REMA) was used to calculate the meta-analytical measures. The decision of whether to report the meta-analysis or not was made depending on the value of I ^2^ .

## Results

**Fig. 1:**
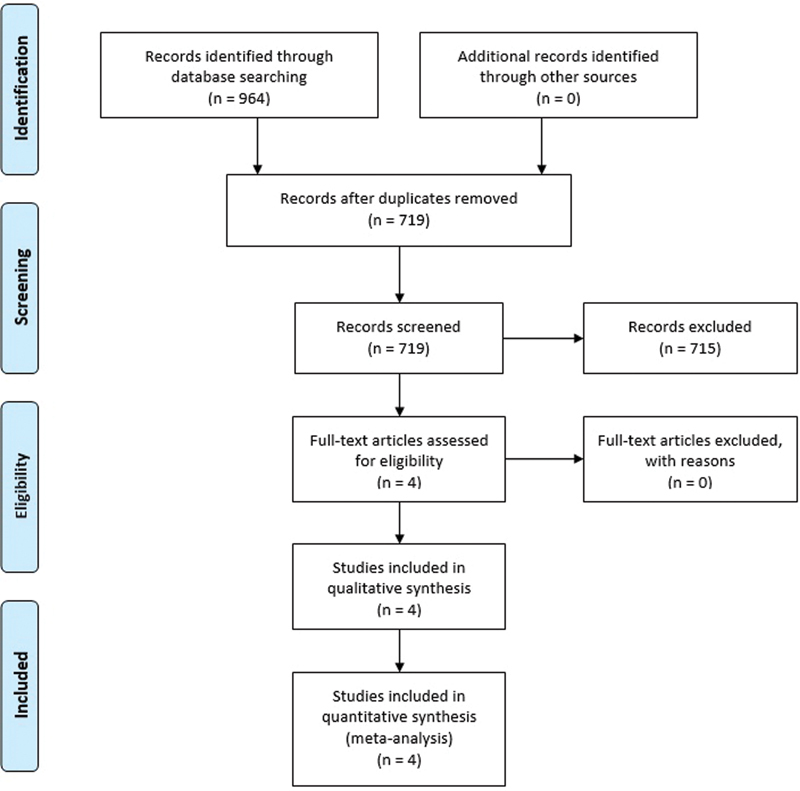
PRISMA flow diagram of the selection process.

The survey returned a total of 964 studies. After removing duplicate records, 719 studies were evaluated by title and abstract, of which 4 were selected for full-text reading. Seven hundred and fifteen studies were excluded because of study design (they were not randomized controlled trials), participant selection (they did not evaluate women in labor), variables analysis (they did not compare continuous versus intermittent catheterization), focus/language of the study (they did not focus on the effects on labor or were written in languages other than Portuguese, English, French, Spanish, or Italian). All full texts assessed for eligibility were selected for inclusion. The selection process is outlined in Fig. 1 .

**Fig. 2:**
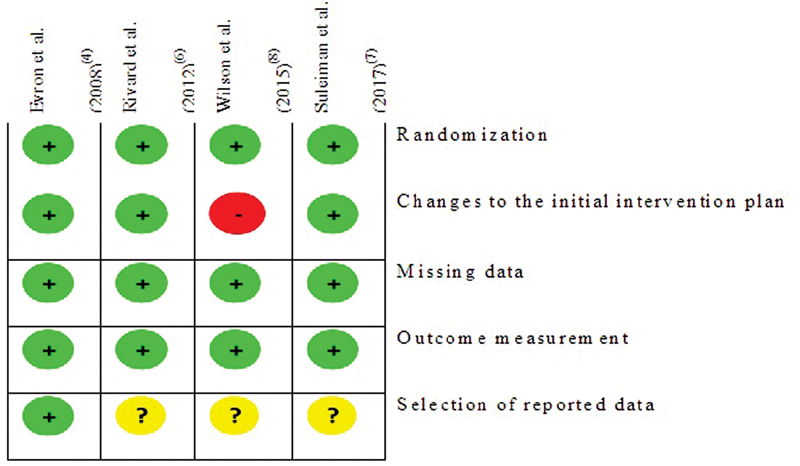
Summary of the risk of bias analysis of the studies included in the review.

The description of the articles is summarized in Fig. 2 . The analysis of methodological quality of the included studies was generally good but revealed some weaknesses. The analysis performed with the RoB2 tool is summarized in Fig. 2 .

To calculate the meta-analytical measure of the duration of the 2nd stage of labor, 2 studies were included (Evron et al., 2008, and Suleiman et al., 2017). 4 7 One of the works (Rivard et al., 2012) 6 was not included because it did not specify the duration of the 2nd stage, and the other because it did not present the standard deviation for the reported labor duration averages (Wilson et al., 2015). 8 Heterogeneity was high (I ^2^ = 85%) and, in this sense, the meta-analytical measure for this outcome was not reported. The duration of the first stage of labor was not evaluated due to the omission of these data by most authors.

## Eutocic Delivery

**Fig. 3:**
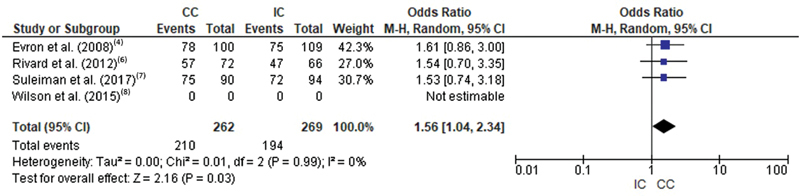
Forest plot of the comparison regarding the occurrence of eutocic delivery in women with intermittent and continuous catheterization.

In assessing the likelihood of eutocic delivery, three studies were included. Only Wilson et al. (2015) 8 did not differentiate eutocic deliveries from operative vaginal deliveries. In this outcome, heterogeneity was low I ^2^ = 0. The meta-analytical measure favored continuous catheterization (OR = 1.56 [1.04, 2.34]; *p* = 0.03). The forest plot of this outcome is shown in Fig. 3 .

**Fig. 4:**
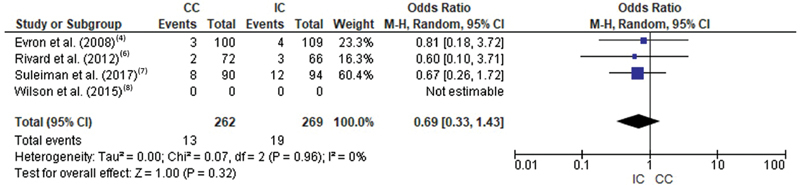
Forest plot of the comparison regarding the occurrence of instrumented delivery in women with intermittent and continuous catheterization.

No differences were found in the number of instrumented deliveries between intermittent and continuous catheterization (OR = 0.69 [0.33, 1.43]; *p* = 0.32), as shown in Fig. 4 .

**Fig. 5:**
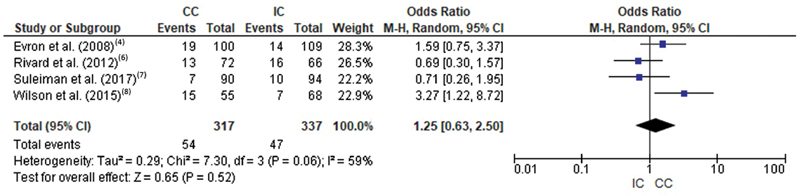
Forest plot of the comparison regarding the occurrence of cesarean section in women with intermittent and continuous catheterization.

No differences were found in cesarean section rate between interventions. In this outcome, the results of the 4 studies were include in the meta-analysis. Heterogeneity was moderate I2 = 59%. The meta-analytical measure found no differences between catheterization methods (OR = 1.25 [0.63, 2.50]; *p* = 0.06). The forest plot is represented in Fig. 5 ( Table 1 ).

**Table 1:** Summary table of the included studies in the review with main findings

Author, Year	Sample characterization	Intervention protocol	Rated outcomes	Results
Evron, 2008 (Evron et al., 2008) 4	n = 209 (IC 109; CC 100) IC group: 25 ± 4 years; 164 ± 6 cm; 74 ± 11 kg; 40 ± 2 weeks; 3 ± 1 cm when epidural; 105 ± 196 minute of membrane rupture; 55% with oxytocic acceleration; newborns with 3,140 ± 450 gr CC group: 26 ± 4 years; 164 ± 6 cm; 78 ± 13 kg; 40 ± 2 weeks; 3 ± 1 cm when epidural; 105 ± 203 minutes of membrane rupture; 67% with oxytocic acceleration; newborns with 3,129 ± 460 gr	IC group: clinical evaluation for the diagnosis of urinary retention every 90 minutes; if urinary retention is suspected - attempted spontaneous urination and bladder emptying if necessary CC group: catheterization after neuroaxis analgesia	Primary outcomes: 2nd stage of labor length; anesthetic dose Secondary outcomes: postpartum urinary retention; postpartum urinary infection	2nd stage of labor longer in the CC group (105 ± 72 minute,) compared with the IC group (75 ± 52 minute). Greater use of anesthetic in the CC group in the first and second stages of labor. Better mobility according to the Bromage scale in the IC group
Rivard, 2012 (Rivard et al., 2012) 6	n = 138 (IC 66; CC 72) IC group: 28.7 years; 2 previous pregnancies, 38 weeks and 2 days, 60% spontaneous deliveries CC group: 27.6 years; 2 previous pregnancies, 38 weeks and 2 days, 50% spontaneous deliveries	After determining the need for bladder emptying IC group: Bladder emptying by catheter every 2–4 hours CC group: catheterization until expulsion period	Primary outcome: time interval until delivery Secondary outcomes: nursing team preference; costs; delivery mode	No significant differences in duration of labor or cost. Nursing team preference for continuous catheterization
Wilson, 2015 (Wilson et al., 2015) 8	n = 123 (IC 68; CC 55) IC group: 26.16 ± 4.76 years; 39.74 ± 1.06 weeks; 4.69 ± 1.33 cm with 91.41% when epidural; 30.99% of induced births; 43.66% with oxytocic acceleration; newborns with 3,445 ± 453.73 gr CC group: 25.87 ± 4.66 years; 39.77 ± 1.22 weeks; 4.52 ± 1.35 cm with 88.23% when epidural; 34.62% of induced births; 46.15% with oxytocic acceleration; newborns with 3,486 ± 445.08 gr	IC group: periodic evaluation (maximum of 6 in 6 hours) and attempted spontaneous urination and intermittent bladder emptying whenever this is not possible (continuous tube placement after 2 catheterizations) CC group: catheterization after neuraxial analgesia	Outcomes Duration of the 2nd stage of labor, incidence of UTI	No differences regarding the duration of the 2nd stage of labor. Higher rate of cesarean sections in the CC group
Suleiman, 2017 (Suleiman et al., 2017) 7	n = 184 (IC= 94; CC = 90) IC group: 27.9 ± 4.5 years; BMI 23.9 ± 4.9 Kg / m2; 39.3 ± 1.3 weeks; 3.4 ± 1.1 cm when epidural; 58.5% of induced births; newborns with 3310.2 ± 423.1 gr CC group: 27.0 ± 4.6 years; BMI 23.8 ± 4.9 Kg / m2; 39.5 ± 1.3 weeks; 3.3 ± 1.2 cm when epidural; 60% of induced births; newborns with 3,264.7 ± 442.2 gr	After epidural and inability to spontaneously urinate IC group: evaluation every 2 hours or when necessary with bladder emptying if unable to urinate or in case of urinary retention CC group: catheterization up to the 2nd stage of labor	Primary outcome: Duration of the 2nd stage of labor Secondary outcomes: type of delivery, duration of the 3rd stage of labor, postpartum hemorrhage, urinary retention, bacteriuria, Apgar score, umbilical artery pH	No differences for included outcomes.

Abbreviations: BMI, body mass index; CC, continuous catheterization; IC, intermittent catheterization; UTI, urinary tract infection.

## Discussion

The purpose of the present review was to assess differences in outcomes related to childbirth between the options available to prevent urinary retention. After evaluating the existing studies and their limitations, the results seem to suggest that both options may be valid.

The risk of bias is globally low. In all studies, randomization was valid, and the allocation was concealed with opaque envelopes. Although the groups were different in one of the studies (Evron et al., 2008), 4 the randomization method was clear, and the differences between groups were described, making it clear that it was random.

There was only one study that changed the initial intervention plan. In this study, at the end of the 2nd need for catheter emptying, the pregnant woman was permanently cuffed, and this happened in 14 of the 55 pregnant women allocated to the intermittent catheterization arm (Wilson et al., 2015). 8

There is no suggestion of missing data in relation to the studied outcomes.

Heterogeneity was high (I ^2^ = 85%), and, as such, it was not considered appropriate to report the meta-analytical measure as statistical heterogeneity would make its interpretation unfeasible. Sensitivity analysis not possible either as only 2 studies were included in the outcome analysis.

Wilson et al. (2015) 8 and Suleiman et al. (2017) 7 found no differences in the duration of the 2nd stage of labor, as did Rivard et al. (2012) 6 in the duration of labor (the latter not specifying the criteria used to start the time counting in labor or differentiating the time of the second stage of labor) between the catheterization types. However, Evron et al. (2008) 4 reported a longer duration of the second stage of labor in the group with permanent catheterization. In this group, there was also a greater need to use anesthetics, and lower mobility, according to the Bromage scale. One possible explanation is related to the fact that the women in this group had a higher body mass index (BMI), which implies that they needed a greater amount of anesthetic and, consequently, had a greater degree of motor block. Moreover, the management of the 2 ^nd^ phase of labor can have a cultural influence, conditioning the obtained data.

Evron et al. (2008), 4 Rivard et al. (2012), 6 and Suleiman et al. (2017) 7 found no differences in the type of delivery and Wilson et al. (2015) 8 did not differentiate operative vaginal deliveries from eutocic deliveries between catheterization types.

The fact that there were more eutocic deliveries in the group of women with intermittent catheterization suggests that in scenarios in which this type of methodology is possible, it should be instituted. However, the preference of users and professionals, which must be an important factor in the decision, and the limited human resources existing in many delivery rooms can be an obstacle to their implementation.

None of the authors reported differences in cesarean section rates, with the exception of except Wilson et al. (2015), 8 who, in their work, described a lower rate of cesarean section in the group with intermittent catheterization. However, they present no explanation for this finding (although the group size could help to explain). Considering all studies, the type of catheterization does not seem to influence the rate of cesarean sections.

In the present review, risks of infection were not assessed. Only one study evaluated the preference of professionals, which is a factor that can affect the practices in the delivery rooms.

None of the included studies analyzed the women's preference for any of the methods, which would be an important factor in decision-making considering the lack of clear advantages of either approach.

Despite the use of four databases considered to be reference in the scientific area of obstetrics, whose scope is high, the use of additional databases could lead to the inclusion of more studies.

## Conclusion

Neuraxial analgesia is associated with a higher urinary retention rate, in some cases, leading to the need for bladder emptying. Urinary retention can produce a mass effect and hinder the descent of the fetal presentation. Catheterization during labor after neuraxial analgesia is not a consensual practice in delivery rooms, and current clinical recommendations do not favor any of the catheterization types (American Society of Anesthesiologists, 2015). 2 The preference and availability of professionals, as well as the preference and the expectation of the parturient, must be considered when deciding whether to carry out the catheterization intermittently or continuously. After reviewing the literature and critically evaluating the four studies performed on the topic, we concluded that there is no clear advantage to either method. However, due to the sample size and the identified bias, the results must be interpreted carefully. Thus, during labor and in low-risk women under neuraxial analgesia, both continuous catheterization and emptying seem to be valid options, although continuous catheterization was associated with a greater occurrence of eutocic births. In the remaining outcomes, there were no differences between catheterization types.
